# Severe Injuries of Former Portuguese Football Players: A Post-Career Burden?

**DOI:** 10.3390/sports13010017

**Published:** 2025-01-09

**Authors:** Eduardo Teixeira, Carlos Silva, Félix Romero, Mauro Miguel, António Vicente

**Affiliations:** 1 School of Sport, Santarém Polytechnic University, 2040-413 Rio Maior, Portugal; csilva@esdrm.ipsantarem.pt (C.S.); fromero@esdrm.ipsantarem.pt (F.R.); mauromiguel@esdrm.ipsantarem.pt (M.M.); 2 Sport Science Department, University of Beira Interior, 6201-001 Covilhã, Portugal; amnv@ubi.pt; 3 Sport Physical Activity and Health Research & Innovation Center (SPRINT), Santarém Polytechnic University, 2040-413 Rio Maior, Portugal; 4 Life Quality Research Centre (CIEQV), Santarém Polytechnic University, 2001-904 Santarém, Portugal; 5 Research Center in Sports Sciences, Health Sciences and Human Development (CIDESD), 6201-001 Covilhã, Portugal

**Keywords:** football (soccer), professional retirement, quality of life, physical domain, career impacts

## Abstract

Background/Objectives: Football players have a high injury risk due to the physical demands of their profession, which can negatively affect their quality of life (QoL) in the long term. The aim of this study is to characterize the severe injuries that former Portuguese football players suffered throughout their professional careers and investigate the resulting impacts on the physical domain of QoL after retirement. Methods: This study includes 84 former Portuguese football players (48.8 ± 8.2 years) who underwent reform between 1988 and 2018 and played in professional leagues (15.2 ± 3.2 years of career duration), namely, first division leagues (n = 43) and secondary division leagues (n = 41). Quantitative and qualitative interview data are used by a validated interview guide and from results of the physical domain obtained by the application of the World Health Organization Quality of Life questionnaire (WHOQOL-Bref). Results: Former players had 2.1 ± 1.5 severe injuries throughout their careers. The most common injuries involve the knee (47.5%) and foot/ankle (17.2%), with the anatomical area of the knee as the most likely to require surgical intervention (60%). The association between tactical-positional status and the incidence of severe injuries is significant, with defenders sustaining less severe injuries throughout their careers. This study found a statistically significant linear model (F(1.82) = 8.089, *p* < 0.006) that explains 7.9% of the variation in the physical domain of QoL (R2ajuste = 0.079). For each severe injury sustained throughout a career, there is an estimated decrease of 2.7 values in QoL perception in the physical domain. Conclusions: The higher the number of severe injuries sustained during a career, the lower the perception of QoL in the physical domain of former players in their post-career. These findings highlight the need to optimize training programs and injury prevention and recovery protocols for long-term QoL improvement in the physical domain of football players.

## 1. Introduction

Football players typically begin their careers at a young age [1] and have confidence in their athletic abilities to achieve professional status [2]. However, the career is short, and injuries are one of the common concerns for players throughout their careers [3,4,5]. The sense of identity anchored in the “physical” body directs this phenomenon towards something equivalent to trauma or chronic illness [6], since the professional status depends on the player’s physical and athletic ability [7,8]. In fact, football is a contact sport played at high speed, which presents a high injury risk [9,10]. Concurrently, this prevalence is also related to the enormous exposure that players have in terms of the number of hours of training and competition [11].

In a study that aimed to provide an update on the current epidemiological numbers in football players competing in all English professional leagues [12], it was found that the estimated incidence is nine to eleven injuries per 1000 h of football-related activity, with the probability of injury being higher in competition (24.29/1000 h) than in training (6.84/1000 h). The incidence of injuries has been increasing over the past two decades [12,13]. Sprains and muscle strains are injuries that have been more prevalent among players [12,14]. In the case of strains, these occur especially at the level of the hamstrings, representing, overall, 39.5% of the total injuries [12]. Concurrently, injuries at the level of the ankles and knees have been highlighted as some of the most problematic for players [15,16]. In the case of the knees, the injuries carry a very significant weight, as practically half result from ligament and meniscus injuries [17,18,19]. For example, in a study conducted in the context of Brazilian football, it was shown that about 66% of former players suffered knee injuries. Of these, 37% needed to undergo at least one surgery, 54% were subjected to injections, and 97% reported experiencing pain at some point in their careers [20]. More than one-third of former players have undergone at least one surgery after the end of their careers, with knee and hip surgeries as the most mentioned. However, when accounting for the treatments or medications resulting from injuries suffered throughout the career, this amount increases substantially [3,14]. In this scenario, it is understandable that osteoarthritis is a problem closely associated with the careers of former footballers [21,22]. The development of this pathology is described in the literature as a factor of lower health perception and quality of life (QoL) in post-career [20,22,23]. It is reinforced that the World Health Organization (WHOQOL Group) defines QoL as “an individual’s perception of their position in life, within the context of the cultural and value systems in which they are embedded, and in relation to their objectives, expectations, norms, and concerns” [24].

Another characteristic associated with football is the impact of the load on specific skeletal bones. Thus, the study of the long-term effects on the bone mineral density of former players also becomes relevant since it has not yet been fully evidenced in research [25], despite the significantly higher tendency among former players in various skeletal regions with a greater incidence of participation in the movement of the sport (e.g., Lumbar spine and femoral neck) [26]. Overload seems to be a decisive factor in the epidemiological issue because some studies conducted during the formative stages of players show that practicing the sport positively affects bone mass during growth [27,28].

The emergence of negative feelings and mental and physical disorders mainly occurs at the moment of transition to post-career, especially in situations where the reasons for the end of career are involuntary [23,29]. Severe injuries emerge here as a determining factor because there seems to be an association between the number of severe injuries throughout the career and the development of mental health [30,31] and physical problems [4,11,12]. The incidence and type of injuries throughout the career highlight possible epidemiological negative impacts in the post-career, which can consequently negatively affect the QoL of former football players. So, the central hypothesis of our study is to verify this trend. In this paradigm, it was deemed pertinent to characterize the severe sports injuries that occurred during the professional career and to assess the consequent impacts on the physical domain of the QoL of former Portuguese players in the post-career stage. So, the following research objectives were defined:

− Quantify and characterize the incidence and anatomical location of severe injuries sustained by former players throughout their careers;
− Quantify and qualify the number and anatomical location of the surgical interventions to which the former players were subjected throughout their career and post-career;
− Analyze the association between the tactical-positional status of former players during their careers and the incidence of severe injuries;
− Verify the relationship between the physical domain of QoL perceived by former players based on the number of severe injuries sustained during their career, the career period, and the competitive level.
− Qualify the physical problems evidenced by former players in the post-career period and relate them to the indicators of QoL perception in the physical domain.

## 2. Materials and Methods

### 2.1. Sample

The sample consisted of 84 former Portuguese football players (age 48.8 ± 8.2 years) that played in professional leagues (15.2 ± 3.2 years of career duration), namely, first division leagues (n = 43) and secondary division leagues (n = 41). Based on the tactical position status that the majority played in their careers, 15 were goalkeepers, 21 defenders, 27 midfielders, and 21 forwards.

A convenience sample was used, taking into account the different regions of residence of former players (Table 1).

Inclusion criteria were former players with at least 8 years of professional experience and a transition to the post-career between 1988 and 2018. To be included in this study, former players had to have at least 3 complete years of retirement, as suggested in the literature [32]. The participants were informed of the anonymity and confidentiality of the data obtained, and this study was approved by the University of Beira Interior Ethics Committee (CE-UBI-Pj-2021-015).

### 2.2. Definition of Variables and Instrument

The research instrument used was the interview guide for the study of impacts of a sports career on the QoL of former Portuguese football players, validated in a previous study [33]. Quantitative and qualitative data were collected specifically from the responses provided by the former players to the questions included in areas 1, 2, and 3 of the interview guide (Table 2).

The data related to the assessment of QoL, particularly in the physical domain, came from a previous study [34], where the World Health Organization Quality of Life questionnaire (WHOQOL-BREF), version validated for the Portuguese language was used [35,36]. In their methodology, all the procedures used for the calculation of QoL were explained, namely, the QoL of the physical domain. The WHOQOL-Bref is used as a generic, multidimensional, and multicultural measure for a subjective assessment of QoL, and the results represent the individual perception in each particular domain [37,38]. The structure of the WHOQOL-Breff is presented, only related to the physical domain (Table 3).

The variables used in the present study are identified and defined:

− Physical domain of QoL: Dependent variable of this study that identifies the QoL values in the physical domain of former Portuguese players in the post-career stage from a previous study [34], obtained through the application of the World Health Organization Quality of Life questionnaire (WHOQOL-BREF) version validated for the Portuguese language [36].
− Severe injuries: Injuries of traumatic origin or overuse that resulted in the functional incapacity of former players [39]. Severe injuries were those that incapacitated the former players from participating in training and competitions throughout their professional careers for a period equal to or greater than 28 days, as suggested in the literature [9,40].
− Anatomical location of severe injuries: The injuries were classified and grouped according to the anatomical location in which they occurred, namely, the foot/ankle, the leg, the knee, the thigh, the hip/groin, the back, the head/face/nose, or other areas, in accordance with what is defined in the literature [13,18].
− Number and type of surgical interventions: Identification of the number of surgeries and/or surgical procedures to which the former players were subjected, during their career or post-career, for the removal or repair of some part of the body or, eventually, to discover the existence of some pathology. For the identification of the type of surgeries, the same criterion of anatomical location described in the previous variable was used.
− Qualification of the physical problems evidenced in the post-career: Testimonies of former players about the problems they exhibit in the post-career at the level of the physical domain.
− Career end period: Determined within a time frame of 30 years (1988–2018). There were two groups formed in order to compare two generations of former players: one for the older players, who finished their careers between 1988 and 2005 (n = 33), and one for the younger players, who completed their careers between 2006 and 2018 (n = 51). The cut value was based on the average number of years of professional abandonment among the 84 individuals surveyed.
− Competitive level: Determined by the divisions in which the former professional players competed throughout their careers. Two levels were considered, the 1st division, which includes former players who played more than 50% of career years in the country’s main league (n = 43), and the 2nd division, which includes players who played more than 50% of career years in the country’s secondary leagues (n = 41).
− Tactical-positional status: Defined by the tactical position that former players identify as the one they primarily played during their career. They are divided into four groups according to their position, namely, goalkeepers (n = 15), defenders (n = 21), midfielders (n = 27), and forwards (n = 21).

### 2.3. Procedures

Semi-structured interviews were conducted through online meetings (ZOOM—Colibri Pro/Licensed/300) between the researcher (first author) and the interviewee (former player), lasting between 55 and 80 min. Participants’ consent was obtained to record audio and/or video recordings, which were then transcribed in a standardized format. A protocol was developed to ensure maximum rigor in data collection, from initial contact with former players to post-interview procedures. The plan included 14 criteria. Specifically, contact selection (i), contact implementation (ii), interview schedule (iii), interview preparation (iv), interview objective explanation (v), interview dynamics explanation (vi), clarification on the treatment and dissemination of results (vii), authorization for interview recording (viii), exposure to the curriculum vitae of the former player’s resume for the confirmation or correction of information (ix), reading and signing informed consent (x), the formal implementation of the interview according to the guidelines in the script (xi), the interruption of the recording at the end of the interview (xii), a moment for the interviewee to add information that they found relevant (xiii), and, finally, addressing personal thanks and greetings (IX). In organizational terms, and, for each of the responses provided, a quantitative and qualitative database was constructed with all the information detailed by variable. It is noteworthy that, for the variable of the QoL assessment in the physical domain, the results calculated and obtained in a previous study were used, exactly with the same sample [34].

### 2.4. Data Processing

The Statistical Package for the Social Sciences was used (IBM SPSS Statistics, version 29) for the analysis of quantitative data. In the characterization of severe injuries and surgical interventions, relative frequencies, percentages, cumulative percentages, means, standard deviations, and minimum and maximum values were used. To evaluate the relationship between the tactical-positional status and the incidence of severe injuries, the chi-square technique was applied. Given the sample size, it was necessary to dichotomize the variable number of severe injuries, using the respective median as the cutoff value. Thus, two groups of former athletes were considered: those who had up to 2 severe injuries during their careers and the group of those who had 2 or more serious injuries. For the identification of significant associations, those with adjusted and standardized residuals greater than 2 in absolute value were considered.

With the aim of verifying the relationship between the perceived QoL (physical domain) by former players and the number of severe injuries sustained during their career, the competitive level, and the career period, a multiple linear regression using the stepwise model was conducted, after verifying the following assumptions:

- Linearity, constant variance, and normal distribution of the residual random variables—through the residual plot;
- Independence of residual random variables—Durbin–Watson Test;
- Non-existence of multicollinearity—Tolerance test.

For the analysis and processing of qualitative information, namely, for the qualification of surgical interventions and for the identification of physical problems evidenced by former players in the post-career, the software QSR International—Nvivo, version 11.0—was used [41,42]. A deductive analysis of the subcategory was carried out, which, through the given responses, gave rise to the respective codes. The descriptive validity was ensured through a triangulation process between the discourse of the former players, the interpretation and conceptual narration of the researchers, and the confrontation with theoretical references.

## 3. Results and Discussion

### 3.1. Characterization of Severe Injuries in Former Football Players

The former Portuguese football players had an average of 2.1 ± 1.5 severe injuries throughout their careers. Of the eighty-four former players surveyed, only seven have not suffered any severe injury (Table 4).

These results confirm the trend of a high incidence rate of injuries during a professional football career, which is in agreement with various investigations in this field [4,9,10,11,17,43]. It can be noted, for example, that 91.7% of former players had at least one severe injury during their careers and that 58.3% suffered at least two severe injuries, which can constitute a risk factor for the development of physical and mental health problems, as mentioned in others studies [20,23,30,31]. These results are not surprising because, if we consider the global incidence of injuries evidenced in a systematic review [44], without being divided by severity level, the average found was 7.8 ± 2.3 injuries per player over the course of their career.

The status of the career is directly dependent on the player’s physical and athletic ability [7] so the physical limitation is equivalent to a trauma or chronic illness [6]. In fact, in the occurrence of this type of phenomenon, players are unable to compete and, most of the time, to train, thus distancing them from their actual work environment. A footballer’s career is relatively short. One previous study with the same sample showed the average career length was 15.2 ± 3.1 years [34]. So, serious injuries may have contributed to a reduction in several months, or even years, in the effective practical time of the careers of various former players, due to the forced breaks in the periods following the injuries.

In this follow-up, and, when observing the data related to the anatomical areas with the highest incidence of injuries among former players, it is noted that almost half of the pathologies occurred at the knee level (Table 5).

These results converge with data from other research, which highlight knee injuries as among the most problematic. This type of injury is the main cause of players’ absence from competitions, with rehabilitation averaging 45 days [44]. Ligament and meniscal problems that occur in this anatomical area are widely mentioned in epidemiological studies with football players [9,17,18,19]. The most affected structures are the anterior cruciate ligament (ACL), the posterior cruciate ligament (PCL), the medial collateral ligament (MCL), and the lateral collateral ligament (LCL), with the ACL as the most commonly affected [3,44].

Injuries at the foot and ankle level were also very common. The results indicate that 17% of the injuries occurred in these anatomical areas that are reported in the literature as very susceptible to sprains [12,14], traumas [14,45], and ruptures, in the case of the Achilles tendon [46,47]. Football is a high-impact sport with enormous stress on the ankle due to explosive and unpredictable movements combined with constant contact between players [44], which justifies the results obtained in this study.

The fourth anatomical area most affected by injuries was at the hip (13%). This is a complex anatomical region that usually presents a diagnostic challenge for doctors [44] because players are greatly affected in the hip and groin area, with common symptoms resulting from the high-intensity impact of the sport.

With a value slightly below what is usually mentioned in the scientific literature, injuries occurring at the thigh level are recorded (5%). This is an anatomical area where, usually, there is a high prevalence of injuries [9,12,15,44]. A possible explanation for this result may be related to the fact that the data collection for this study is limited to severe injuries; that is, it is possible that their prevalence is more related to less severe injuries that occur frequently but have downtime of less than a month [9,39]. It is also important to highlight the high percentage of unspecified injuries (12.7%).

From the content analysis, it was found that the majority of severe injuries sustained in this dimension were at the anatomical areas of the shoulders, arms, forearms, and hands. There was also a report of a severe injury occurring in the genital area. Resulting from the 181 severe injuries sustained by the former players throughout their careers, it was found that the average number of surgical interventions was 1.6 ± 1.6 per player. Nevertheless, it should be noted that 28.6% did not undergo any surgery (Table 6).

The high number of surgeries performed by former players during their careers is in agreement with some studies that indicate football as one of the sports with the highest incidence of surgeries [17,45,48]. From Table 7, it is noted that the majority of surgical interventions occurred at the anatomical location of the knee (60%), followed by operations at the foot/ankle location (12.3%).

These results align with the conclusions of some studies that indicate that massive injuries in football players at the level of the lower limbs are responsible for a large part of the surgical interventions occurring in these anatomical areas [4,23,45].

Already after the end of their careers, only ten former players underwent surgical interventions, with two of them repeat cases (Table 8). The results indicate a much lower trend of post-career surgery compared to the results of other studies [14,17], which indicate that more than a third of former players undergo at least one surgery after the end of their career.

Of the total surgical interventions after the end of their careers, four were on the knee, one on the foot, one on the spine, and four originated from other causes not directly related to injuries sustained in football. Thus, through content analysis, an attempt was made to infer the reasons and particularities of these surgical interventions. Starting with the former players who underwent surgical interventions to resolve physical problems directly related to the impact of their professional careers, the following accounts are presented:

“*I could barely walk. The meniscus was a problem, so I really had to have surgery. On top of that, I really enjoyed continuing to run and play with my young players. I really liked executing and showing how it’s done, so it was a way to minimize the weaknesses felt*.”

(former player 5)

“*I had surgery for a cervical hernia. It was completely related to my career in football, I have no doubts. The surgical procedure was well resolved, it took a long time, but that was due to the national health system, until reaching the operation*.”

(former player 13)

“*When I stopped playing, I already had limitations in my knee, and I worsened that injury at the age of 40 in a recreational game. I took a hit to the knee and it ended up causing this injury to my cruciate ligaments. I ended up having a pretty good recovery*.”

(former player 27)

“*As a player, I had a slight knee problem, and it worsened due to my activity as a goalkeeper coach. I’ve already had surgery and it’s causing me a hassle*.”

(former player 76)

“*I stopped playing football and ended up having to undergo surgery twice for the sequelae I had during my career. Surgery on one foot and another on the knee, both of which went well and minimized some of the pain I had*.”

(former player 82)

As can be seen, only one of the surgical interventions was not related to the anatomical area of the knee, which supports the trend reported in two studies in the field stating that the anterior cruciate ligament rupture, medial collateral ligament rupture, and medial meniscus rupture are the injuries that most impact players’ lives in the medium and long term [40,44], and, consequently, more surgeries may occur in this anatomical area of the knee in the post-career phase. Regarding the surgical interventions mentioned due to causes external to the professional career, the following testimonies were obtained:

“*I had surgery for a duodenal ulcer. It appeared suddenly, not being related to the career*.”

(former players 3)

“*I had my gallbladder removed while I was taking the coaching course. It went smoothly, I just have to be careful with my diet nowadays*.”

(former player 10)

“*At 41, I had my first heart attack. I needed to undergo surgery, and then, later on, I had my second heart attack. The fact that I had this type of professional activity and lived it with great intensity and passion made me not take much care with rest and recovery, and then this happened to me. But I’m on medication, I’m doing great, thanks to God*.”

(former player 21)

“*I had an appendectomy*.”

(former player 41)

“*I had a surgical medical intervention, but it was because I was playing around. Broke my clavicle*”

(former player 51)

Epidemiological events are observed that are perfectly analogous to those occurring in individuals of the general population; that is, the former players encountered casual health events that have no relation to the risk factors inherent to the professional career. Despite the small number of cases mentioned, it is important to highlight that, if the impacts of the career are negative in the long term, former players in post-career may be doubly exposed to health and QoL risks, not only due to physical problems resulting from the career but also to factors associated with aging and diseases that may arise over the course of life.

### 3.2. Tactical-Position Status and Incidence of Severe Injuries

The association between the tactical-positional status of former players during their careers (goalkeepers, defenders, midfielders, and forwards) and the incidence of severe injuries was significant (Table 9). The analysis of the adjusted and standardized residuals reveals that this association is only significant for defender players, who have significantly incurred fewer severe injuries than other playing positions throughout their careers (Table 10).

In football, the tactical positions of players have differentiated specificities, particularly in terms of the impact of internal and external load. The different demands required for each playing position are one of the factors related to the incidence of sports injuries [49,50,51], which can explain the differences found in this research by itself. In fact, several studies indicate that players who act as central defenders cover shorter distances in training and competition and, concurrently, have fewer high-intensity actions [49,51,52,53]. This indicates that this tactical position is tendentially less impactful from a physical standpoint. In a recent study [52], it was found that central defenders have a significantly lower work rate in sprints, decelerations, accelerations, and changes in direction compared to players in other positions. For example, when analyzing high-intensity efforts, forwards, central midfielders, and side defenders emerge as the most demanding positions [49,52,53] and, consequently, are more susceptible to contracting muscle injuries. External load indicators are, therefore, strictly related to footballers’ injuries [22,44], which supports the trend observed in the association between positional status and the incidence of injuries in this sample of former Portuguese football players.

### 3.3. Impact of Severe Injuries on the Physical Domain of QoL in the Post-Career Period

When analyzing the results related to the impact of serious injuries on the physical domain of the QoL of former players in the post-career period, it appears that the linear model found is statistically significant [F (1.82) = 8.089, *p* < 0.006] and explains 7.9% of variations in the physical domain of QoL (R2adj = 0.079).

Of the initially proposed independent variables, only the number of severe injuries significantly explains the perception of QoL in the physical domain of former players. (Table 11).

The variables related to the competitive level (first division and secondary divisions) and the career period (1988–2005 and 2006–2018) were excluded (Table 12).

The equation found is as follows:

- Physical domain of QoL = 86,636–2626 severe injuries during the career.

This means that each severe injury sustained during a career decreases the perception of QoL in the physical domain by 2.626 points. The variables of competitive level and career period do not have explanatory power over the perception of QoL in the physical domain. The results are in line with a previous study [34] that, for the same sample of former players, found a statistically significant relationship (R = −0.300; *p* = 0.006) between the physical domain of QoL and the number of severe injuries of former players during their professional career. Thus, it can be asserted that severe injuries have a negative impact on the perception of QoL in the medium and long term. This conclusion is aligned with other studies in the epidemiological field [44,45], which indicate that this negative impact occurs at both the professional and amateur player levels, so the concern of injury prevention and recovery programs and protocols should be directed with the same level of attention to different competitive levels.

For a deeper understanding of this paradigm, a qualitative analysis was conducted on the type of physical problems experienced by former players in their post-career, particularly those who suffered serious injuries during their careers. There are some testimonies:

“*My problem is in the knee, which prevents me from doing certain things I used to enjoy. Today I already have difficulties running, so I choose to do other activities like walking and cycling*.”

(former player 2)

“*The physical aspect always suffers a lot with the career. I’ve been taking Voltaren my whole life. I’ve been injected many times to relieve the pain. What I feel are the repercussions of all this*.”

(former player 5)

“*I have some problems, especially at the hip. A very significant wear in the hip area caused by football. About 2 years ago, during a routine exam, I was detected with cardiac changes that required me to undergo a catheterization. I assume that I could have been identified as a professional athlete, they never detected it, but what is certain is that I have heart conditions. I have a reduction in physical activity and I have to live with it. I take some medication to control the problem*.”

(former player 9)

“*The only things that continue to manifest are the result of the various injuries I have had throughout my career. I think I’m going to have these pains for the rest of my life, aren’t I?! We are talking about a part of the body that does all the balancing up and down, the body equilibrium, and I think I will always have these knee pains*.”

(former player 17)

“*I think it’s related to the long career I had, where I spent my body for many years, every now and then I have knee pain, I think I got a permanent injury in my knee*.”

(former player 23)

“*Nowadays I have pain because I made the career I did and, well, there are players who can always be at the top and don’t have physical problems because maybe they are better prepared in some way. The body’s genetics is different and everything is related. Talking about me, yes. Sometimes I joke and tell my wife that I don’t know if I’ll last much longer because there are things I feel nowadays that I think a person twice my age doesn’t feel*.”

(former player 25)

“*It left me with some physical ailments. I can clearly feel my knee with some deficiency. Now I think I limp a little. I no longer bend my leg fully, I also no longer do the exercises I should have done, but well, we have to keep enduring*.”

(former player 32)

“*Yes, I have two hernias, one in the cervical and another in the lumbar. Both are related to football. The follow-up was very poor. It’s a shame because we suffer a lot with sports and many injuries remain*“.

(former player 36)

“*The knee injuries have atrophied my leg. I have to be very careful. I like to play tennis, but I have to be cautious*.”

(former player 38)

“*Yes, I have it in my knee and foot, but it’s a matter of knowing how to live with it. But yes, it left marks, but it’s a matter of knowing how to manage, at least I can manage. It has repercussions because it is a high-level, very aggressive sport, and therefore it always leaves marks*.”

(former player 41)

“*Yes, I think it’s normal, you reach an age close to 50 and joint problems become more pronounced. I have a lot of difficulties with my knees at certain times. The problems I have now are related to the injuries from my career. The relationship with my knees is very different from that of my friends who didn’t play football*.”

(former player 51)

“*I feel various pains, mainly in my collarbones and one of my ankles. I don’t always have it, but at certain times, during a change of season or when it’s a bit more humid, I feel a lot of pain. They are clearly related to the ailments I have been experiencing throughout my career*.”

(former player 59)

“*Many (referring to pains)! Back pain, shoulder pain … even those things we were just talking about, the sprains when the cold comes... I have already arthritis cases*.”

(former player 62)

“*My health problems end up being related to football, stemming from degenerative wear of the bony part of the knees mainly, spine, disc hernias, and are linked to the hip joint. I should already have a hip prosthesis, I don’t have cartilage. I am postponing the intervention*.”

(former player 67)

The accounts are revealing of the physical problems that former players face in their post-career. Generally, they are related to the physical impacts of the professional career and, particularly, to the severe injuries they had. Limitations, problems, and physical pains are repeatedly mentioned and, naturally, influence the QoL of these individuals, as evidenced in another study in the field [45]. As an example above, we observed cases related to osteoarthritis, which is a problem widely associated with the careers of former footballers [19,20,21,22,54] and described in the literature as a factor of lower health perception and quality of life in post-career [17,20,21,22,23].

It is clear that the number of severe injuries negatively impacts the physical domain of the QoL of former players. Nevertheless, more studies will be needed to deepen the understanding of the causes and effects of the mentioned health problems. Physical impacts arise not only exclusively from the occurrence of severe injuries but also, for example, from issues related to the mechanical overload imposed by the profession over the years of practice [27,28]. Concomitantly, there are the effects associated with the aging of the former players themselves, which can trigger, in themselves, physical and health problems common to the population of the same age group that did not engage in high-level sports, although several studies unanimously point out that a professional career in football significantly increases the likelihood of developing long-term physical problems [17,45,55]. Just as a reference, three reported cases are presented that seem to have no relation to the impacts of the professional career and the severe injuries associated with it:

“*Just anxiety attacks, but they have a strong impact on my life. I’ve ended up in the hospital twice because of this problem*.”

(former player 12)

“*About two years ago, I had a heart problem, although it was due to a throat inflammation, I had a mild heart attack. I think, it was not related to the career. But a few months ago, I started having a lot of pain in my neck, and after going to the doctor, he said it was nerve entrapments. I don’t know if it has anything to do with the pandemic, from being more tense, or if it could be from playing football, especially because I used to head the ball a lot*.”

(former player 50)

“*I had a systemic cardiovascular accident 4 years ago, but fortunately, that was all, I had no after effects. It may have been derived from the career or it may not have been*.”

(former player 82)

These examples can be considered in future research lines.

Considering the results obtained, it is important to define guidelines for support programs and services during the professional career and post-career period as suggested in several studies [56,57,58,59]. In the physical domain, and to mitigate the risk and impact of the injuries, it is mandatory to provide a robust clinical program, with strong technical and scientific guidelines. This allows efficiency in the diagnosis of injuries, the quality of clinical intervention and the respective treatment, and the rehabilitation and integration of players in the training and competition process, which will bring to players positive consequences in terms of physical quality of life in post-career.

## 4. Conclusions

Former Portuguese football players had an average of 2.1 ± 1.5 severe injuries throughout their careers. The knee is the anatomical area with the highest incidence of severe injuries (47.5%), followed by injuries at the foot/ankle level (17.2%). Of the 181 serious injuries sustained by the former players throughout their careers, the average number of surgical interventions was 1.6 ± 1.6 per player. The majority of the surgeries occurred in the anatomical area of the knee (60%). The association between the tactical-positional status of former players during their careers and the incidence of serious injuries showed that players who played as defenders suffered significantly fewer severe injuries than goalkeepers, midfielders, and forwards. The analysis of the results regarding the impact of severe injuries on the physical domain of QoL of former players in the post-career phase indicated that the number of severe injuries significantly explains the perception of QoL in the physical domain of former players; that is, for each severe injury sustained during the career, the former player decreases the perception of QoL in the physical domain by 2.7 points. The qualification of the physical problems evidenced by former players in their post-career reinforced this evidence. The variables related to the competitive level and the career period did not have explanatory power over the perception of QoL in the physical domain.

The results highlight the importance of monitoring the professional career in football at the level of identifying risk factors that expose players to injuries. Simultaneously, it highlights the need to continue optimizing training programs and injury prevention and recovery protocols that are crucial for improving the QoL in the physical domain of former football players during their careers and, consequently, minimizing possible negative impacts in the post-career period.

## Figures and Tables

**Table 1:** Regions of residence of former players (Nuts II).

	N	Percentage
North	15	17.9
Center	25	29.8
Lisbon and Tejo Valley	27	32.1
Alentejo	6	7.1
Algarve	3	3.6
Madeira and Azores	6	7.1
Outside Portugal	2	2.4
Total	84	100.0

**Table 2:** Areas and categories used based on the interview guide.

Area 1: Biographical Data	Area 2: Professional Career	Area 3: Transition to Post-Career
Category 1: personal data	Category 2: personal data Category 4: epidemiological pathway	Category 7: post-career epidemiological pathway

**Table 3:** Facets of the physical domain of QoL in the structure of WHOQOL-Breff.

	Domain	Facets
WHOQOL-BREF	Physical (6-Q3) + (6-Q4) + Q10 + Q15 + Q16 + Q17 + Q18	Pain and discomfort Energy and fatigue Sleep and rest Mobility Activities of daily living Medication or treatment dependence Work capacity

**Table 4:** Frequency of severe injuries throughout the career.

	Frequency	Percentage	Cumulative Percentage
without severe injuries	7	8.3	8.3
1 severe injury	30	35.7	44.0
2 severe injuries	20	23.8	67.9
3 severe injuries	13	15.5	83.3
4 severe injuries	8	9.5	92.9
5 severe injuries	3	3.6	96.4
6 severe injuries	2	2.4	98.8
7 severe injuries	1	1.2	100.0
Total	84	100.0	

**Table 5:** Anatomical location where severe injuries occur.

	Frequency	Percentage
Foot/ankle	31	17.2
Leg	7	3.86
Knee	86	47.51
Thigh	9	4.97
Hip	13	7.18
Back	5	2.76
Head, Face, and Nose	7	3.86
Others	23	12.71
Total	181	100.0

**Table 6:** Frequency of surgical interventions during the career.

	Frequency	Percentage	Cumulative Percentage
Without surgical interventions	24	28.6	28.6
1 surgical intervention	24	28.6	57.1
2 surgical interventions	18	21.4	78.6
3 surgical interventions	10	11.9	90.5
4 surgical interventions	3	3.6	94.0
5 surgical interventions	3	3.6	97.6
6 surgical interventions	2	2.4	100.0
Total	84	100.0	

**Table 7:** Surgical interventions during the career (based on anatomical location).

	Frequency	Percentage
Foot/ankle	16	12.3
Leg	4	3.1
Knee	78	60
Thigh	4	3.1
Hip	5	3.8
Back	5	3.8
Head, Face, and Nose	4	3.1
Others	14	10.8
Total	130	100.0

**Table 8:** Frequencies of surgical interventions in post-career.

	Frequency	Percentage	Cumulative Percentage
Without surgical interventions	74	88.1	88.1
1 surgical intervention	8	9.5	97.6
2 surgical interventions	2	2.4	100.0
Total	84	100.0	

**Table 9:** Chi-square tests.

	Valor	df	Asymptotic Significance (Bilateral)
Pearson’s Chi-squared	10.269 ^a^	3	0.016
Likelihood ratio	10.507	3	0.015
Linear association	0.629	1	0.428
N valid cases	84		

^a^. 0 cells (0.0%) expect a count less than 5. The expected minimum count is 6.61.

**Table 10:** Cross-tabulation frequency of severe injuries and tactical-positional status.

			Tactical-Positional Status				Total
			Goalkeeper	Defenders	Midfielders	Forwards	
Frequency of Severe Injuries	Up to 2 severe injuries	Counting	4	15	12	6	37
		Adjusted Residuals	−1.5	2.9	0.1	−1.6	
	2 or more severe injuries	Counting	11	6	15	15	47
		Adjust	1.5	−2.9	−0.1	1.6	
Total		Count	15	21	27	21	84

**Table 11:** Multiple regression coefficients and collinearity statistics.

Coefficients ^a^								
Model		Unstandardized Coefficients		Standardized Coefficients	t	Sig.	Collinearity Statistics	
		B	Erro	Beta			Tolerance	VIF
1	(Constant)	86.636	2.365		36.633	<0.001		
	Severe injuries during the career	−2.626	0.923	−0.300	−2.844	0.006	1.000	1.000

^a^. Dependent variable: physical domain of QoL (TWD1).

**Table 12:** Excluded variables ^a^.

Model		Beta In	t	Sig.	Parcial Correlation	Collinearity Statistics		
						Tolerance	VIF	Minimum Tolerance
1	Competitive level	0.118 ^b^	1.119	0.267	0.123	0.995	1.005	0.995
	Career period	−0.035 ^b^	−0.333	0.740	−0.037	0.996	1.004	0.996

^a^. Dependent variable: physical domain of QoL (TWD1). ^b^. Predictors in the model: (constant), severe injuries during career.

## Data Availability

The study database can be provided by the corresponding author.
